# An Update on Advanced Diagnostic Imaging in Dentistry

**DOI:** 10.3390/diagnostics12051041

**Published:** 2022-04-21

**Authors:** Dario Di Nardo, Alessio Zanza, Francesco Pagnoni, Edit Xhajanka, Luca Testarelli

**Affiliations:** 1 Department of Oral and Maxillo-Facial Sciences, Sapienza University of Rome, 00161 Rome, Italy; rodolfo.reda@uniroma1.it (D.D.N.); francesco.pagnoni22@gmail.com (F.P.); luca.testarelli@uniroma1.it (L.T.); 2 Department of Prostethic Dentistry, Faculty of Dentistry, Medical University of Tirana, 1001 Tirana, Albania; edit.xhajanka@umed.edu.al

In recent years, CBCT has proved to be extremely widely used and widespread in dentistry. Thanks to the high availability, the reduced radiant dose, the possibility of managing the FoV acquisition and the definition of the images, the reduced execution times, and the compatibility with subsequent surgical, prosthetic, aesthetic and orthodontic analysis and design software, CBCT represents the ideal imaging technique in dentistry [1,2].

Recent evidence shows that even the most complex orthodontic treatment plans can be performed with adequate software on the basis of a single diagnostic examination, without the need to prescribe several as was practiced up to now, and always having a diagnostic examination available can also be extremely useful and reliable for the dental treatment plan as a whole [3,4,5]. In the complex evaluation of the orthodontic treatment plan and the final rendering, in addition to both precise and expensive extraoral scanners, it is possible to obtain remarkable results even with simple extraoral photographs that are commonly used to evaluate orthodontic therapy follow-up [2,6].

Furthermore, 3D exams reworked by specific software allow for extremely complex and greatly simplified evaluations, but it is also important, in light of the diagnostic confirmation obtained with 3D, to validate the use of those still prescribed 2D exams [7]. Three-dimensional diagnostic examinations, if prescribed with small FoVs, can be integrated with 2D exams, or can be considered useful for finding prognostic indices in 2D radiography [6,7].

Moreover, the application of optical coherence tomography for the evaluation of resin infiltration for the repair of enamel white spots has recently been proposed in the literature, confirming the results obtained from this recently introduced treatment [8]. The evolution of the different imaging techniques in conservative dentistry allows for, as in the case of synchrotron ATR-FTIR chemical imaging, useful and powerful approaches to the microspectroscopic diagnostics of molecular composition in the hybrid sound dentin/dental composite interfaces and materials, including ones developed with the use of biomimetic strategies [9].

It is precisely for this reason that increasing attention must be paid to the radiant dose, with prescriptive accuracy as regards radiographic examinations, and increasing attention paid to radiation-free examinations, the use of which must be greatly encouraged [10,11]. In particular, great attention is paid to MRI (magnetic resonance imaging), which has specific sequences and suitable devices, although also having long acquisition times and reduced availability; thus, it seems to date to be in many ways superimposable to complex 3D radiographic examinations. In light of the diagnostic tests with ionizing radiation prescribed to verify outcome therapy and subsequent follow-up, imaging methods without ionizing radiation must be carefully investigated in the near future [12,13,14].

The goal to be achieved is that, in the near future, the major limitations of these devices are overcome and that ionizing radiation-free diagnostic tests, such as MRI or ultrasounds, become commonly prescribed for diagnostic and prognostic purposes [10,15].

In this regard, recent research on the use of ultrasound in dentistry shows how its application is now possible and even easier. For this specific method, despite the limitation of being operator-dependent, and therefore, with the need for a certain learning curve on the part of the clinician, the ease of having this device, which is not bulky and is simple to use, encourages the development of studied components for the oral cavity [10,16].

The goal for this imaging method, for the foreseeable future, is that it replaces the low dose 2D intraoral radiographic examination for the evaluation and monitoring of bone lesions [16].

Current results and trends in the literature show us how traditional 3D radiographic examinations with increasingly reduced radiation doses are the most prescribed, and how these new evolutions are being studied, with applications of specific technologies or with the reduction of biological damage caused by them [10,11,14].

Radiation-free diagnostic tools attract a lot of researchers’ attention; an excellent index for the future development of equipment and software that make them more easily usable in daily clinical practice [10,14].
